# Climate-Driven Range Extension of *Amphistegina* (Protista, Foraminiferida): Models of Current and Predicted Future Ranges

**DOI:** 10.1371/journal.pone.0054443

**Published:** 2013-02-06

**Authors:** Martin R. Langer, Anna E. Weinmann, Stefan Lötters, Joan M. Bernhard, Dennis Rödder

**Affiliations:** 1 Steinmann Institut für Geologie, Mineralogie und Paläontologie, Rheinische Friedrich-Wilhelms Universität, Bonn, Germany; 2 Biogeography Department, Universität Trier, Trier, Germany; 3 Department of Geology and Geophysics, Woods Hole Oceanographic Institution, Woods Hole, Massachusetts, United States of America; 4 Zoologisches Forschungsmusem Alexander Koenig, Museum Koenig, Bonn, Germany; Université Paris Sud, France

## Abstract

Species-range expansions are a predicted and realized consequence of global climate change. Climate warming and the poleward widening of the tropical belt have induced range shifts in a variety of marine and terrestrial species. Range expansions may have broad implications on native biota and ecosystem functioning as shifting species may perturb recipient communities. Larger symbiont-bearing foraminifera constitute ubiquitous and prominent components of shallow water ecosystems, and range shifts of these important protists are likely to trigger changes in ecosystem functioning. We have used historical and newly acquired occurrence records to compute current range shifts of *Amphistegina* spp., a larger symbiont-bearing foraminifera, along the eastern coastline of Africa and compare them to analogous range shifts currently observed in the Mediterranean Sea. The study provides new evidence that amphisteginid foraminifera are rapidly progressing southwestward, closely approaching Port Edward (South Africa) at 31°S. To project future species distributions, we applied a species distribution model (SDM) based on ecological niche constraints of current distribution ranges. Our model indicates that further warming is likely to cause a continued range extension, and predicts dispersal along nearly the entire southeastern coast of Africa. The average rates of amphisteginid range shift were computed between 8 and 2.7 km year^−1^, and are projected to lead to a total southward range expansion of 267 km, or 2.4° latitude, in the year 2100. Our results corroborate findings from the fossil record that some larger symbiont-bearing foraminifera cope well with rising water temperatures and are beneficiaries of global climate change.

## Introduction

Sea surface temperature is a key environmental predictor that affects the biogeographic distribution of many organisms. Global climate change is likely to alter the range of areas potentially suitable for habitation [1]–[4]. Among the predicted effects of rising temperature is the range expansion of species into areas where they previously did not exist [5]–[8]. The expansion of species ranges along their cooler boundaries appears to be a prominent consequence of the global warming trend [7], [9]. A rapidly increasing number of studies have shown “fingerprints” of recent climate-driven changes in various biological systems. This includes range shifts of species towards higher latitudes, higher elevation and earlier springtime phenologies [9]–[12]. To date, however, only a limited number of studies have addressed rates of range shifts in marine biotas [13].

Our studies concern the distribution and biogeographic range expansion of unicellular, larger symbiont-bearing foraminifera in modern oceans [4], [14]. Larger symbiont-bearing foraminifera have a circum-tropical distribution and are indicative of warm tropical and subtropical waters [14], [15]. Temperature has long been considered as the primary factor regulating their latitudinal distribution [14]. For the majority of these foraminifera, the lower temperature limit is 18 to 20°C [14]. Relatively low temperatures are tolerated by species of the genus *Amphistegina* and their distributional range is currently delimited by the 13.8° winter isotherm [4], [16], [17].

Among the larger symbiont-bearing foraminifera, amphisteginids are of particular interest because they display the widest latitudinal ranges in all oceans [14]. Today, amphisteginids have been found as far as 40° North and 31° South [14], [18]. They are among the most conspicuous and ubiquitous foraminifera on coral reefs and tropical carbonate shelves [14], where they often have been referred to as living sands [19]. As key carbonate producers [20]–[22], amphisteginids contribute significantly to carbonate substrate stability, growth of reefal structures, and habitat formation [20]–[24]. Recent studies have shown a widening of the tropical belts with far-reaching changes for oceans, ecosystems, and the biosphere [25]–[27]. A distributional range expansion of amphisteginid foraminifera due to global warming could trigger substantial changes in ecosystem functioning (e.g. changes in species diversity, carbonate production, impact on native biota [4]).

Recently, amphisteginid foraminifera were shown to expand their biogeographic range in the Mediterranean where a rapid progression northwestwards now shows them closely approaching the Adriatic and Tyrrhenian seas [4], [18], [28], [29]. In addition, their increasing abundance and rapid proliferation of invasive amphisteginids were shown to impair the dynamic equilibrium of established foraminiferal biotas, ultimately replacing diverse assemblages by rapidly spreading monocultures [4].

To date, the overwhelming majority of studies on organismal range shifts in response to climate change focus on terrestrial species [30]. In contrast, comparatively few studies have addressed range shifts in marine systems [31]–[35] although the impact of climate-driven range expansion is considered the “next frontier” in climate change research [36]. Understanding, monitoring, and predicting range expansions are, therefore, vital for effective management and conservation [37], [38]. Consequently, there is a pressing need to improve our ability to predict these phenomena. This requires the deployment of modeling approaches that can be successfully utilized at a range of spatial scales. Here we apply a species-distribution model (SDM) to assess potential range expansions of *Amphistegina* spp. under current and future climate conditions (for the years 2050, 2100) along the eastern coast of southern Africa. The coastline from equatorial Somalia along Kenya, Tanzania, and Mozambique to Cape Town, in South Africa, displays a distinct latitudinal sea-surface temperature gradient that is ideally suited to apply SDM and to project range shifts in the future. In addition, the Indian Ocean has been undergoing a pronounced multidecadal warming trend [39] that is likely to affect the biogeographic ranges of species living along the eastern coastline of Africa.

Species distribution models have been applied previously to project range expansions of amphisteginids in the northern hemisphere including the Mediterranean Sea [4]. In this study, we provide the first modeling approach for foraminifera from localities of the southern hemisphere to assess the rates of range expansions under rapid environmental change. The model uses an environmental envelope of information from localities where amphisteginids are currently known to occur. The physical niche constraints were compiled from all presently available data containing the environmental conditions for population dynamics. The environmental variables that define the current niche of east African amphisteginids then were used to develop correlative models to extrapolate potential occurrences at sites where the environmental constraints are projected to match physiological constraints under current and future conditions. We also calculated the rate of amphisteginid range expansion along latitude based on historical and recent occurrence records, and compared them to rates currently observed for amphisteginids in the Mediterranean. Rates of latitudinal range expansion in *Amphistegina* were then compared with rates recorded in recent reviews on the expansion of terrestrial and marine taxa [10], [13], [40], [41]. Using this modeling approach, we project the result on climate scenarios for the years 2050 and 2100, and compute the extent of potential range expansion and the probable speed of future range shifts.

## Materials and Methods

### Species Records and Environmental Data

**Figure 1:**
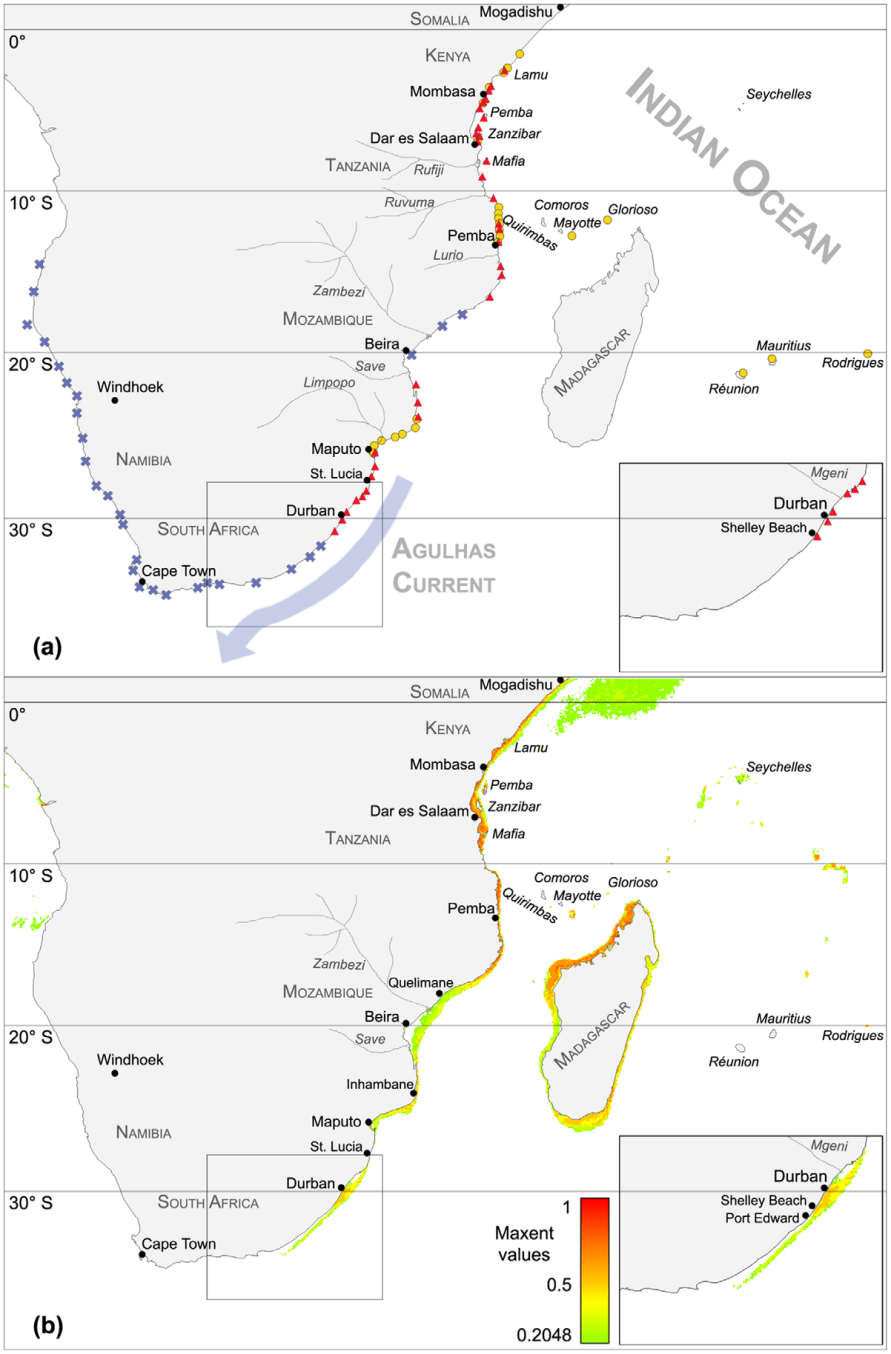
*Amphistegina* occurrence records and SDM for under current climate conditions. (A) *Amphistegina* occurrence records used to create the Species Distribution Model. Sites marked with a triangle are new records collected by this working group between 2004 and 2012, those with circles are sites from published data, and those with an x are sites where *Amphistegina* were absent (our data). Insert shows the southernmost occurrence record of *Amphistegina* at Shelley Beach (30.84°S) south of Durban. (B) Species distribution model for *Amphistegina* spp. with 2012-occurrence records (triangles) and potential distribution under current climate conditions as projected by the SD-model. Habitat suitability is indicated by individual Maxent values ranging from high (1) to extremely low (0.2). The model projects potential settling sites expand to Port Edward (31°S) south of Shelly Beach. Note that habitat suitability south of Shelly Beach is deflected away from the coast by the southward flowing Agulhas Current.

The species distribution modeling and range expansion analyses of *Amphistegina* is based on an extensive sample set that was collected recently (2004–2012) between equatorial sites of Somalia/Kenya, and Cape Town, in South Africa (no specific permits were required for the described field studies). We compiled all amphisteginid species occurrences and collected additional citations from primary literature and review papers. The biogeographic survey covers a latitudinal range between 1.2° S at Somalia and 34° S at Cape Town (Figure 1A). Literature occurrences were extracted from historical and recent studies [42]–[67]. Occurrences of amphisteginid foraminifera were recorded from a total of 118 sites, and include 82 sites from our own sampling and 36 literature records. Our own samples were taken by scuba-diving or by a Van Veen grab at depths between 0 and 200 m, and include both live and dead foraminifera. Occurrence records of dead individuals were only included when the test(s) did not show any signs of transport. All records are situated within unique grid cells derived from DIVA-GIS [68].

Environmental data were obtained from the BIO-ORACLE dataset designed for species distribution modeling [69]. The data package contains a global set of 23 variables obtained through remotely sensed and *in situ* measured data. Raster grid-cells of BIO-ORACLE have a resolution of 5 arcmin or 9.2 km. We used the mean sea surface temperature (°C), diffuse attenuation (an indicator of water turbidity, m^−1^), the minimum chlorophyll a content (mg/m^3^), the annual sea surface temperature range (difference between maximum and minimum, °C) and the maximum photosynthetically active radiation (Einstein m^−2^ day) as biologically relevant predictor sets for SDM modeling.

The SDM scenarios for range expansion of *Amphistegina* for the year 2050 and 2100 are based on predictions provided by the Fourth Assessment Report of the Intergovernmental Panel on Climate Change [70]. We used the response of the 30-year average SST between 2070–2099 and 1961–1990 as an approximation for the rise in ocean temperature until 2100. For the model for 2050, we adopt a 50% decrease of the 2100 temperature approximation. The winter prediction was presumed to conform to SST_min_ and the summer prediction was used for SST_max_. The projected climate change datasets then were applied to our georeferenced grid-cell format.

### Computation of the SDM

We used Maxent 3.3.3 k for SDM modeling and projections onto future climate conditions [71], [72]. The program uses a grid-based, machine-learning algorithm following the principles of maximum entropy [73]. It is a presence-only method, generating pseudo-absences from a defined background, ideally covering areas potentially colonizable for the taxon [74]. The Maxent modeling begins with a uniform distribution and successively fits the distribution to the data (occurrence records and environmental variables). By iteratively permuting and varying the input variables, Maxent repeatedly tests the predictive capability and improves the model. This is measured as log likelihood or “model gain” that records increasing distances from the uniform distribution. A full description and details of the procedure can be found in Elith *et al.* [75].

To predict the potential and future amphisteginid distribution, a total of 10,055 random background points were automatically selected by Maxent from the biogeographic range of *Amphistegina* spp. along the eastern coast of Africa. Maxent then predicts the suitability of a habitat, representing the potential distribution of the taxon. For clarity, the logistic output format with suitability values ranging from 0 (unsuitable) to 1 (optimal) was used [76]. The probability of the taxon’s presence at sites with “typical” environmental conditions is set to 0.5 by default [75]. The modeling process was performed with 30 replicates and the average predictions across all replicates were used for further processing.

### Model Evaluation

The quality of the SDM provided by Maxent can be tested by calculating the Area Under the Curve (AUC) and refers to the Receiver Operation Characteristic curve [72]. The AUC reassess the ability of the model to distinguish presences from (pseudo-) absences. Occurrence records were randomly split into training (70%) and test samples (30%). This non-parametric method is recommended for ecological applications [77], [78]. Values of AUC range from 0.5 for models with no better than random predictability to 1.0 for models supplying perfect SDM prediction. According to the classification of Swets [79], AUC values >0.9 describe “very good”, >0.8–0.9 “good”, and >0.7–0.8 “useful” discrimination ability. The continuous probability surfaces of the SDMs were subsequently converted into presence/absence maps using the “Equal training sensitivity and specificity logistic threshold” as recommended by Liu *et al.* [80]. The impact of individual environmental predictors for the resulting model is specified as the percent contribution of every variable. Additional evaluations are provided by the permutation importance, which displays the drop in AUC values (normalized to percentages) when the values of every variable on training presence and background data are randomly permuted. Furthermore, a jackknife test is implemented in Maxent, which allows the analysis of the predictability potential of individual variables. The model is repeatedly created by using variables in isolation to examine how well the results fit the known model gain (both on training and test data) and the AUC values. To assess the importance of individual predictors, each variable then is omitted, and model gain and AUC value are evaluated. A decrease in model gain results in an approximation of the model to the uniform distribution.

## Results

Computed data provide “very good” AUC values (AUC_training_: 0.9703; AUC_test_: 0.9513) for our SDM. The lowest Maxent value obtained at the training records is 0.0239. Analysis of the relative contributions of environmental variables reveals the following hierarchy in descending order of explanatory power (Table 1): Mean sea-surface temperature (SST_mean_) has the highest explanatory power with 41.78%, followed by mean diffuse attenuation (DA, 25.11%), minimum chlorophyll (CHLO, 23.07%), sea-surface temperature range (SST_range_, 6.99%), and maximum photosynthetically available radiation (PAR, 3.04%). The permutation importance of individual variables reveals a similar picture (Table 1). The jackknife tests show that omitting SST_mean_ from the model results in the sharpest drop in model gain and AUC values, followed by DA, CHLO, SST_range_, and PAR (Table 2). When used in isolation, DA and SST_mean_ provided the best results (Table 2).

**Table 1:** Variable contribution and permutation importance for predictors used during model training.

	Contribution [%]	Permutation importance [%]
**Mean sea-surface temperature**	41.78	47.19
**Mean diffuse attenuation**	25.11	30.97
**Minimum chlorophyll a**	23.07	16.26
**Maximum photosynthetically available radiation**	3.04	1.39
**Sea-surface temperature range**	6.99	4.18

Note high values for mean sea surface temperature, mean diffuse attenuation, and minimum chlorophyll indicating their importance as prime factors regulating the distribution and habitat suitability of amphisteginid foraminifera.

Model iterations using the maximum winter surface temperatures show an increasing range expansion, but not to the extent of the model presented here. Mean SST accounts for nearly 42% of the modeled effect, while SST range contributes only 7% to the variation. Hence the model presented exhibits the maximum range extension that can be predicted but it is realized that amphisteginids may not fully occupy this potential niche in the future as they do today.

Pilot studies involving the present range prediction of amphisteginids along the African coast using minimum chlorophyll α values parallel the empirical biogeographical distribution. This parameter was retained in the SDM with the understanding that maximum chlorophyll α values would impact the foraminiferal ranges.

**Table 2:** Results of the Jackknife test for training and test data.

	Training gain	Test gain	AUC values
**Species distribution model for ** ***Amphistegina*** ** spp.**	**2.2215**	**2.1563**	**0.9513**
**Model without variable:**			
*Mean sea-surface temperature*	1.4824	1.3361	0.8897
*Mean diffuse attenuation*	2.1529	2.1274	0.9509
*Minimum chlorophyll a*	2.1804	2.148	0.9505
*Maximum photosynthetically available radiation*	2.1852	2.1442	0.951
*Sea-surface temperature range*	2.0586	2.038	0.9459
**Model with variable in isolation:**			
*Mean sea-surface temperature*	0.5427	0.6459	0.8018
*Mean diffuse attenuation*	0.6616	0.5953	0.7959
*Minimum chlorophyll a*	0.6413	0.5856	0.7923
*Maximum photosynthetically available radiation*	0.3108	0.2944	0.6908
*Sea-surface temperature range*	0.2637	0.3123	0.7012

Note strong decrease in gain and AUC values for mean sea-surface temperature, sea-surface temperature range, and mean diffuse attenuation when omitted from the SDM for *Amphistegina*. When variables are used in isolation the values are most similar to the original gain and AUC for mean sea surface temperature, mean diffuse attenuation, and minimum chlorophyll. This indicates their important role in regulating the biogeographic distribution of amphisteginid foraminifera.

All amphisteginid occurrence records modeled in the area are displayed in Figure 1A. The current biogeographic range covers the area from tropical sites at the equator off Somalia (1°N at Somalia) southward along the coastlines of Kenya, Tanzania, Mozambique, and south to Shelley Beach in South Africa at 31°S. The distance from the equator at Somalia to the southernmost occurrence record covers ∼3410 km (1° = 110 km). Offshore island occurrence records of amphisteginid foraminifera from the southwestern Indian Ocean include sites off Madagascar, Reunion, Mauritius, Rodrigues, the Seychelles [54], Glorioso Islands [48], and Mayotte [45].

Figure 1B displays the potential distribution for amphisteginid foraminifera as computed from SDM under current climate conditions. The potential distribution of the genus correlates well with the actual biogeographic range. The distribution model reveals, however, that amphisteginid foraminifera may potentially expand their coastal range occurrences further south to Port Edward (31°S), which is located 18.34 km south from the southernmost record at Shelley Beach (30.84°S). The potential distribution thus exceeds the known realized distribution by only a few kilometers. The model also shows that the areas of highest suitability comprise the coastal regions between 1°N (Mogadishu) and 17°S (Quelimane). Lower habitat suitability values are indicated for the areas between the Zambezi River Delta (18.1°S), Beira (20°S), and the Save River Delta (21°S).

**Figure 2:**
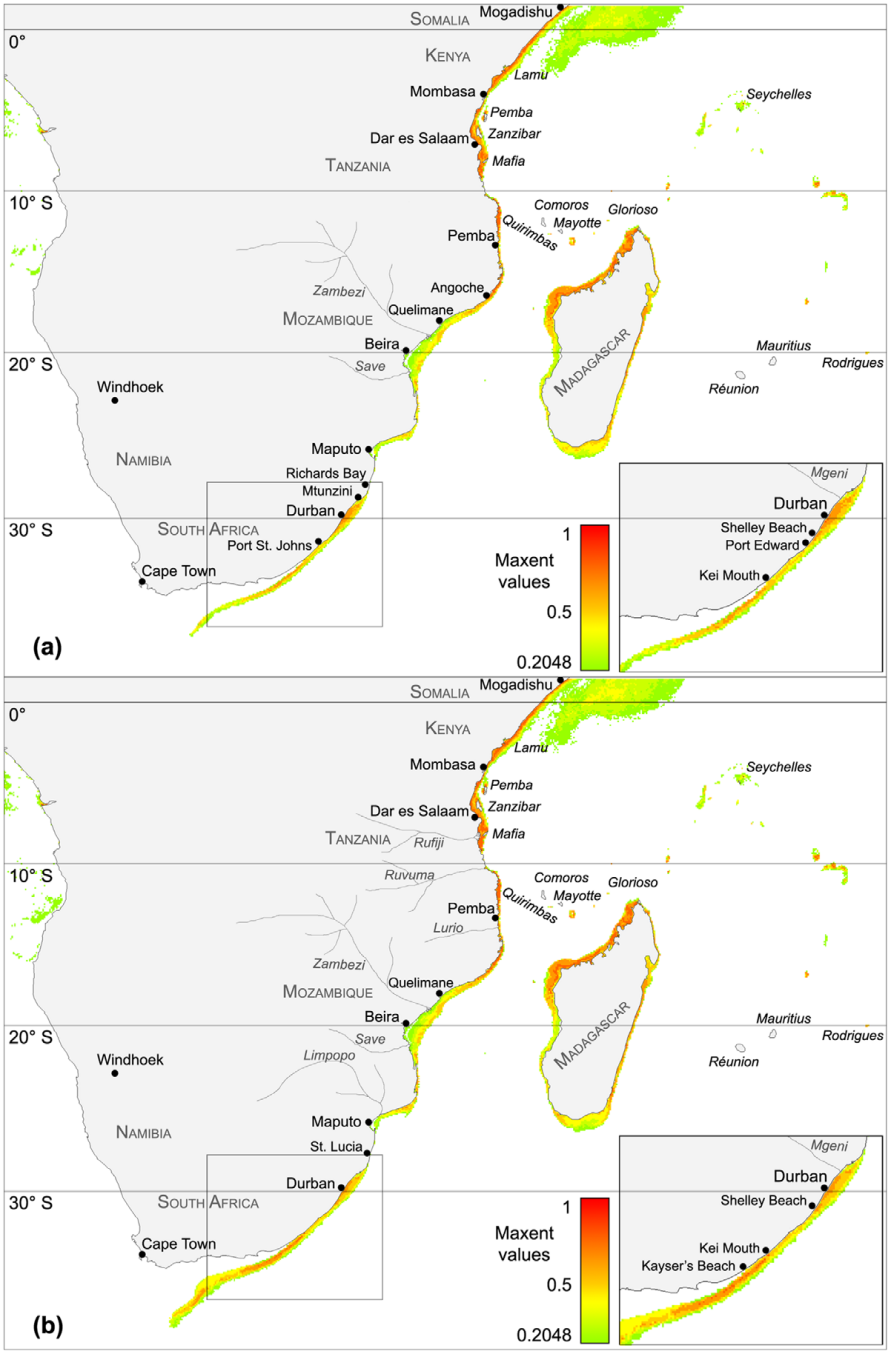
Species distribution models for the years 2050 and 2100. (A) Species distribution model for *Amphistegina* spp. projected under climate conditions for the year 2050. Habitat suitability is indicated by individual Maxent values ranging from high (1) to extremely low (0.2). Note that the model projects potential settling sites expand southward to Kei Mouth (32.65°S) south of Port Edward (inset). The model also projects increasing habitat suitability around Durban and between Angoche and the area north of Maputo. Note that habitat suitability south of Kei Mouth is deflected away from the coast by the southward flowing Agulhas Current. Range expansion to higher latitudes and habitat suitability is mainly governed by climate-driven temperature increases. (B) Species distribution model for *Amphistegina* spp. projected under climate conditions for the year 2100. Habitat suitability is indicated by individual Maxent values ranging from high (1) to extremely low (0.2). Note that the model projects potential settling sites expand southward to Kayser’s Beach Mouth (33.22°S) south of Durban (inset). The model projects a general increase of habitat suitability from the equator to the area north of Maputo and south of Durban. Note that habitat suitability south of Kayser’s Beach is deflected away from the coast by the southward flowing Agulhas Current.

The SDM computed under climate conditions projected for the year 2050 projects a substantial southward shift of habitat suitability for amphisteginid foraminifera (Figure 2A). The model projects a coastal range expansion south to Kei Mouth, located at 32.65°S. The prognosticated range extension to Kei Mouth is an additional 1.641° ( = 182.68 km) south of the potential range of *Amphistegina* at Port Edwards, and 201.12 km south of the current southernmost occurrence record at Shelley Beach. Climate conditions projected for the year 2050 indicate that SST increases from 23.26°C to 24.51°C for the Shelley Beach location. In the 2050 model, the areas of highest suitability comprise the coastal regions between 1°N (Mogadishu) and 17°5S (Quelimane), and 29°S (Mtunzini) and 31.4°S (Port St. Johns). Areas of low suitability values continue to be between the Zambezi River Delta (17.5°S), Beira, and the Save River mouth (21°S).

The SDM computed under climate conditions projected for the year 2100 predicts a temperature rise of 2.5°C and an additional range expansion of amphisteginid foraminifera (Figure 2B). The model indicates a southward expansion along the coast of South Africa down to Kayers`s Beach, located at 33.22°S. The modeled range expansion to Kayers`s Beach marks an additional latitude extension of 0.5754° ( = 64.05 km), compared to the potential range of *Amphistegina* for the year 2050. The model computed for climate conditions for the year 2100 indicates a total southward range expansion of 265.07 km ( = 2.4°), when compared to the current distribution limit of amphisteginid foraminifera.

In the model for the year 2100, highest suitability areas for amphisteginid foraminifera are between the equatorial coastal regions off Somalia (Mogadishu at 1°N) and Quelimane near the northernmost end of the Zambezi Delta (18°S). Low habitat suitability values were computed for coastal areas between major rivers, Zambezi and the Save (18°S –21°S), and around the Maputo Harbor area (26°S).

### Biogeographic Range Expansions

Occurrence records of amphisteginid foraminifera indicate that the current biogeographic range covers the coastal area from the equator at Somalia 1°N to Shelley Beach (31°S) in South Africa, and a multitude of offshore islands in the Indian Ocean (Figure 1A). The biogeographic range of east African amphisteginid foraminifera attests that species of this genus have a tropical to subtropical distribution delimited by the 14°C winter isotherm [14].

East African amphisteginid foraminifera were reported first by d’Orbingy [81], Wright [82], [83], and Möbius [42] from tropical locations off Madagascar, Mauritius, and the Seychelles. Later, Heron-Allen & Earland [45] recorded three species of *Amphistegina* from the nearshore Kerimba Archipelago between 11.5°S and 13°S. Records of amphisteginids from the coast of Kenya (between 3° and 4°S) were published by Pereira [54], Banner & Pereira [84], and Lévy *et al*. [57]. Amphisteginid foraminifera also were reported by Braga [46], Moura [48] and Perry [64] from the area between Inhambane (24°S) and Maputo (26°S). The southernmost occurrence records of *Amphistegina* published to date, are from the St. Lucia (28.23°S) and Mgeni Estuaries (29.48°S) in South Africa (28.23°S) [59], [61]. Their presence here was attributed to the warm temperatures that were carried southward by the warm Agulhas Current [60]. Extensive sampling in the St. Lucia region in 1972 and 1973 by Phleger [51], [52] did not reveal any amphisteginid foraminifers, suggesting that the amphisteginid records of Wright *et al.* [61] from the same location in 1990 were recent arrivals at that time. South of Durban, at 29.53°S, occurrences of living amphisteginid foraminifera previously were not reported even though extensive sampling was undertaken [44], [62], [63], [85]–[87]. Belderson’s report [88] of amphisteginid foraminifera from Durban Bay could not be verified by Albani [85], but suggests that this taxon has repeatedly tried to colonize habitable environments along the coast. Recent and extensive sampling campaigns (2004–2005) conducted by this working group reveal numerous new amphisteginid occurrences as far south as Shelley Beach (30.84°S). This indicates a southward biogeographic range extension over ∼1.3° latitude [ = 145 km, from the Mgeni Estuaries [60] to Shelley Beach (Langer, unpubl. data]) between 1987 and 2005, equivalent to a range extension of ∼ 8 km year^−1^.

The range expansion projected by the SDM under climate conditions of the year 2050 (Figure 2A) prognosticates the occurrence of amphisteginid foraminifera south to Kei Mouth (located at 32.65°S). The predicted range extension to Kei Mouth is an additional 201.12 km south of the current southernmost occurrence record at Shelley Beach (30.84°S). This would constitute an average range shift of ∼5.29 km year^−1^ if the range shift is realized in the year 2050. The model for 2050 also projects a general increase in habitat suitability for amphisteginid foraminifera along the east coast of Africa (Figure 2A). Suitability is particularly increased from the equator at Somalia, along the coast of Kenya and Tanzania, down to Angoche in Mozambique (16.2°S). The SDM also shows an increase in habitat suitability from Richards Bay (28.4°S) to Port Edward (31.0°S) in South Africa.

For climate conditions predicted for the year 2100 (Figure 2B), the species distribution model suggests a total range expansion over 2.38° latitude southwards to Kayers`s Beach at 33.22°S (∼242 km). If realized in 2100 and referenced to the current southernmost occurrence record at Shelley Beach, the marine range expansion of amphisteginids would occur at an average rate of ∼2.75 km year^−1^. The *Amphistegina* SDM generated for climate conditions in the year 2100 shows increasing habitat suitability over large stretches of coastline from the equator at Somalia to Kayers`s Beach at 33.22°S in South Africa. Areas of lower habitat suitability are indicated for the regions around the major river mouth off the Rufiji, Rovuma, Lurio, Zambezi, Save, and Limpopo rivers. Low values of habitat suitability are also displayed around Beira, and between Maputo in Mozambique and Richards Bay in South Africa.

## Discussion

Average global temperature has increased over the past century (0.74±0.18°C) and overall global warming is predicted to continue to rise between 2.0 and 4.5°C over the next 100 years [89]. Global warming and the extension of climate belts are likely to allow substantial range expansion for species with tropical or subtropical origins. This study reports the first SDM approach to project potential range expansion of foraminifera under current and future climate conditions. The computed SDM values affecting the biogeographic range reveal that sea surface temperature had the highest explanatory power among individual variable contributions (51%), followed by mean diffuse attenuation (25%) and minimum chlorophyll (23%). This agrees well with 1.) observations in the Mediterranean Sea where temperature has been identified as the key agent governing the range expansion of amphisteginid foraminifera [4] and 2.) with recent findings that temperature alone can readily predict the large scale geographic structure of shallow biogeographic schemes with 53–99% accuracy [90]. The Mediterranean range shift of amphisteginid foraminifera is attributed to the ongoing warming trend in the northern hemisphere. Research conducted on foraminifera from the east coast of Africa provides a baseline chronology illustrating an analogous spread of amphisteginids towards higher latitudes in the southern hemisphere.

Based on historical occurrence records, the range shift of coastal amphisteginid foraminifera was calculated to occur at an average rate of ∼ 8 km year^−1^ between 1987 and 2012. The average rate prognosticated under climate conditions for the year 2050 and 2100 was computed to continue at an average rate of ∼ 5.29 km year^−1^ (for the year 2050) and at ∼ 2.75 km year^−1^ until the year 2100. However, it needs to be noted that the actual rate of marine range expansions in our case is likely to be higher. This is because the latitudinal calculations underestimate the true distances along east-westward oriented coastlines. In addition, the southward-flowing, warm Agulhas Current is deflected away from the coast by the cold Benguela Current, diverting potentially suitable habitats from coastal regions into open ocean territory.

The calculated range-expansion rates for *Amphistegina* from the east coast of Africa are at the lower end of average range shifts currently known for marine plankton, invertebrates, and vertebrates [13]. Sorte *et al.* [13] have calculated that the average rate shift in marine organisms occurs at 19.0 km year^−1^. They also noted that the vast majority of range shifts in marine species were in poleward direction, consistent with global climate change scenarios. Rate shifts computed for amphisteginid range expansion in the northern hemisphere (Mediterranean Sea) were found to occur at an average rate of 12.5 km year^−1^, concordant with expansion rates of other Lessepsian migrants in the Mediterranean [91]. Higher range-expansion rates computed for the Mediterranean Sea, however, appear not to be related to sea surface current velocities when compared to the southwestward flowing Agulhas current along the coast of east Africa, which is among the fastest flowing ocean currents (peaks speed up to 2 m/s). The computed range-expansion rates for amphisteginid foraminifera are up to an order of magnitude faster than rates for terrestrial range shifts (0.61±0.24 km year^−1^) [13]. This observation commonly is attributed to the more open nature of marine versus terrestrial populations [13], [92]. Because of their abundance and high reproductive rates [93], [94], foraminifera are generally expected to adapt fast to warming climates. The comparatively low rates of range expansion recorded in this study highlight the importance of incorporating additional information about range-limiting factors in native communities, environmental tolerances, and species interactions.

Water temperature previously has been invoked as the major factor controlling the latitudinal extension of amphisteginid foraminifera [4], [14], [16]. In particular, Langer & Hottinger [14] have demonstrated that the ranges of larger symbiont-bearing foraminifera are limited by the minimum winter temperature extremes. Indeed, sea surface temperature data compiled from the Indian Ocean Thermal Archive (IOTA) show a significant late 20th Century Indian Ocean warming (0.5–1.0°C) [39], [95]. Although warming has affected all oceans [96], [97], rising temperatures are more pronounced in the Indian Ocean and recently reached their highest values in 120,000 years [98], [99]. The observed southward directed range expansion of thermophile amphisteginids along the eastern coast of Africa makes the ongoing warming trend the most likely agent facilitating the taxon’s current range expansion.

Paleontological evidence indicates that amphisteginid and other larger foraminifera cope particularly well with rising water temperatures and widening of the tropical/subtropical climate belt [100]–[107]. Miocene specimens are reported from Poland and the Vienna Basin at paleolatitudes between 48–50°N [101]. Cretaceous and Eocene records [101] of larger symbiont-bearing foraminifera show a range extension from the equator to almost 50°N and 40°S [101], [106]. In contrast, larger symbiont-bearing foraminifera from modern oceans are mostly limited between 40° North and 30° South, or the 20°C surface-water isotherms during the summer. The latitudinal range extensions during the Miocene, Eocene, and Cretaceous were attributed to higher surface-water temperatures during warmer climates similar to those affecting the poleward extension of current regimes [101], [106]. The range extensions and mass abundances of larger foraminifera during climate periods with increased atmospheric CO_2_ [108]–[110] suggest that foraminifera are, potentially, beneficiaries of climate-driven temperature changes (e.g., nummulit, orbitolinid or alveolinid mass deposits, see also [22], [111], [112]. Global changes are not solely restricted to temperature increases. In addition to warming, the pH in oceans is currently decreasing due to the increased load of atmospheric carbon dioxide compared to pre-industrial times. This decline in pH, or ocean acidification (OA), likely will impact all marine organisms but is expected to most significantly affect organisms that secrete calcium carbonate hard parts (skeletons, shells, tests). Thus, while warming may permit pole-ward expansion of habitat for some species, OA may cause this expansion to be more challenging than if warming alone were occurring. In the case of *Amphistegina gibbosa*, laboratory experiments indicate that the survival of this species is not negatively impacted after 6-week incubation in 1000 or 2000 ppm pCO_2_ compared to ambient control incubations [113]. Additionally, some specimens also reproduced in the enriched CO_2_ incubations, suggesting that amphisteginid dispersal, fitness, and range changes may not be significantly hampered by OA. Similar findings were obtained from experiments on *Amphistegina radiata* [114].

The SDM computed under current climate conditions is closely congruent with the occurrence records of modern amphisteginids indicating that a few easily acquired oceanographic parameters are sufficient to predict the taxon’s biogeographic range. This robust, first-order link between the environmental parameters and biogeographic ranges indicate that model-based predictions may be applied to project large-scale system-level changes. Our analyses also suggest that the exceptionally detailed and well-preserved fossil record of foraminifera may be used to reconstruct the general paleoceanographic structure of ancient shallow seas. Such an application permits the use of fossil data in projected model scenarios of shelf and coastal ecosystems in a warm future.

### Effects on Ecosystems

As noted, amphisteginid foraminifera are among the most prolific foraminiferal species and contribute significantly to the stabilization of reefal frameworks worldwide [20]–[22]. They are prominent producers of calcium carbonate within the world’s oceans where they often add more than 1 kg of CaCO_3_/m^2^/year to reef carbonate sediments [20]–[22]. In some of the east African reefs, amphisteginid foraminifers frequently represent up to 50% or more of the foraminiferal fauna (Langer, unpubl. data), implying that they play a prominent role in reef ecosystems.

The range expansion of amphisteginid foraminifers in the Mediterranean Sea was shown to lead to a drastic reduction of foraminiferal species diversity, increased carbonate production, substrate modifications and, at some sites, to the establishment of amphisteginid monocultures [4], [115]. Because of their abundance, ubiquity and appearance in monocultures and as prominent carbonate producers with substrate modifying capabilities, amphisteginid foraminifera can be considered ecosystem engineers in the sense of Jones *et al.* [116], [117]. While the immediate impact of such changes appears to be obvious, the resilience of ecosystems to the disruptive forces of key invaders remains to be determined. Given their prominent environmental role, rapid biogeographic range expansion, and impact on native ecosystems, amphisteginid range expansion and invasion into new territory are likely to trigger changes in ecosystem functioning [4]. With predicted environmental suitability increasing southward, further studies monitoring environmental changes and modification in community structure along the eastern coast of southern Africa are required.

In conclusion, the Indian Ocean is undergoing a warming trend that affects the biogeographic range of the native biota and ecosystem functioning. Amphisteginid foraminifera are among the key species that currently are expanding their range and rapidly progress southwestwards, closely approaching the coastline of Port Edwards in South Africa. Temperature has been identified as the most important physical oceanographic variable controlling their spatial distribution, congruent with analogous observations in Mediterranean amphisteginids [4] and numerous other marine ectotherms [3], [13], [90], [118], [119].

The computed rates of range shifts (between 2.75 and 8.0 km year^−1^) are at the lower end of average marine range-expansion spectrum and project a total range shift of 2.4° latitude for the year 2100. Examination of the rates of range shift and modeling potential range shift under future climate conditions provides a first approximation that helps to identify sites and magnitudes of potential impact. This, in turn, is vital to assess climate-change impact on coastal marine biotas and will be of use in directing monitoring efforts. It is anticipated that climate warming and the widening of the tropical/subtropical belt will foster the poleward migration of amphisteginid and other larger symbiont-bearing foraminifera. Modern and paleontological evidence of foraminiferal range expansions indicate that some species of symbiont-bearing foraminifera benefit from rising temperatures and become predominant producers of calcium carbonate under conditions of global climate warming.
